# Evaluation of OPEN Zinc Finger Nucleases for Direct Gene Targeting of the *ROSA26* Locus in Mouse Embryos

**DOI:** 10.1371/journal.pone.0041796

**Published:** 2012-09-06

**Authors:** Mario Hermann, Morgan L. Maeder, Kyle Rector, Joseph Ruiz, Burkhard Becher, Kurt Bürki, Cyd Khayter, Adriano Aguzzi, J. Keith Joung, Thorsten Buch, Pawel Pelczar

**Affiliations:** 1 Institute of Laboratory Animal Science, University of Zurich, Zurich, Switzerland; 2 Molecular Pathology Unit, Center for Cancer Research, and Center for Computational and Integrative Biology, Massachusetts General Hospital, Charlestown, Massachusetts, United States of America; 3 Transposagen Biopharmaceuticals, Inc., Lexington, Kentucky, United States of America; 4 Institute for Experimental Immunology, University of Zurich, Zurich, Switzerland; 5 Institute of Neuropathology, University Hospital Zurich, Zurich, Switzerland; 6 Department of Pathology, Harvard Medical School, Boston, Massachusetts, United States of America; 7 Institute for Medical Microbiology, Immunology, and Hygiene, Technische Universität München, Munich, Germany; Ohio State University Comprehensive Cancer Center, United States of America

## Abstract

Zinc finger nucleases (ZFNs) enable precise genome modification in a variety of organisms and cell types. Commercial ZFNs were reported to enhance gene targeting directly in mouse zygotes, whereas similar approaches using publicly available resources have not yet been described. Here we report precise targeted mutagenesis of the mouse genome using Oligomerized Pool Engineering (OPEN) ZFNs. OPEN ZFN can be constructed using publicly available resources and therefore provide an attractive alternative for academic researchers. Two ZFN pairs specific to the mouse genomic locus *gt(ROSA26)Sor* were generated by OPEN selections and used for gene disruption and homology-mediated gene replacement in single cell mouse embryos. One specific ZFN pair facilitated non-homologous end joining (NHEJ)-mediated gene disruption when expressed in mouse zygotes. We also observed a single homologous recombination (HR)-driven gene replacement event when this ZFN pair was co-injected with a targeting vector. Our experiments demonstrate the feasibility of achieving both gene ablation through NHEJ and gene replacement by HR by using the OPEN ZFN technology directly in mouse zygotes.

## Introduction

Mouse lines carrying genes that have been disrupted (knocked-out) or modified (knocked-in) by homologous recombination (HR) are important tools that are widely used in biomedical research. Such lines are generated by gene targeting in mouse embryonic stem (ES) cells and subsequent morula aggregation or blastocyst injection of positive clones to generate chimeric animals [1], [2]. Despite several improvements aimed at shortening the time frame of this approach [3] and considerable efforts of consortia such as EUCOMM, KOMP, or NorCOMM to target all mouse genes [4], [5] engineering the mouse genome remains expensive, time-consuming and is often plagued by technical problems such as genomic stability of ES cells and subsequent difficulties in obtaining germline competent chimeras.

Zinc finger nucleases (ZFNs) have been conceived as an alternative means of selectively altering the eukaryotic genome [6]. ZFNs are custom endonucleases that generate double-strand breaks (DSBs) in their target DNA sequence. Each monomer consists of 3 to 6 DNA-binding zinc finger modules and the endonuclease domain of *Fok*I [7]. The zinc finger modules specify a binding site of 9 to 18 bps thus allowing the design of ZFN pairs specific for “half-sites” with a total potential specificity of up to 36 bp in length. ZFN pairs can be produced by modular assembly of one-finger [8], [9] or two-finger modules [10], [11] with predefined binding characteristics or by selection-based methods such as the Oligomerized Pool Engineering (OPEN) protocol developed by the Zinc Finger Consortium (http://www.zincfingers.org) [12], [13]. OPEN relies on a bacterial two-hybrid selection system [12] to identify ZFNs from combinatorial zinc finger libraries, which exhibit high activities and specificities for their intended target sites. OPEN ZFNs have been used to efficiently modify endogenous genes in zebrafish [14], plants [15], [16], and human somatic [12] and pluripotent stem cells [17], [18]. Using the sequences of a large number of OPEN ZFNs, a selection-free approach known as Context-Dependent Assembly (CoDA) was also recently described that yielded active ZFNs in zebrafish and plants with a success rate of approximately 50% [19], [20].

DSBs caused by ZFN activity can be repaired by either error-prone non-homologous end joining (NHEJ), the dominating DNA repair mechanism in most eukaryotes [21], or by high fidelity HR [22]. ZFN-induced mutations caused by mutagenic NHEJ have been used to generate knockout zebrafish through microinjection of ZFN mRNAs or ZFN expression constructs directly into embryos [11], [14], [23] and similar approaches were also used to manipulate the genomes of rat [24]–[26] and mouse [27] following microinjection of zygotes. In a further refinement, HR-mediated gene targeting was achieved by co-injection of ZFN mRNAs together with targeting constructs into mouse and rat zygotes [28], [29]. Most of these experiments were carried out with ZFNs generated by proprietary technology of Sigma-Aldrich/Sangamo BioSciences Inc.. By contrast, ZFNs generated using the OPEN platform and other publicly available assembly kits [30], [31] can be constructed directly by the end user.

Here, we tested two OPEN ZFNs designed to target the mouse *gt(ROSA26)Sor* (*ROSA26*) locus. Our findings demonstrate that OPEN ZFNs can be used to achieve gene ablation through NHEJ and gene targeting by HR directly in mouse zygotes.

## Results

**Figure 1:**
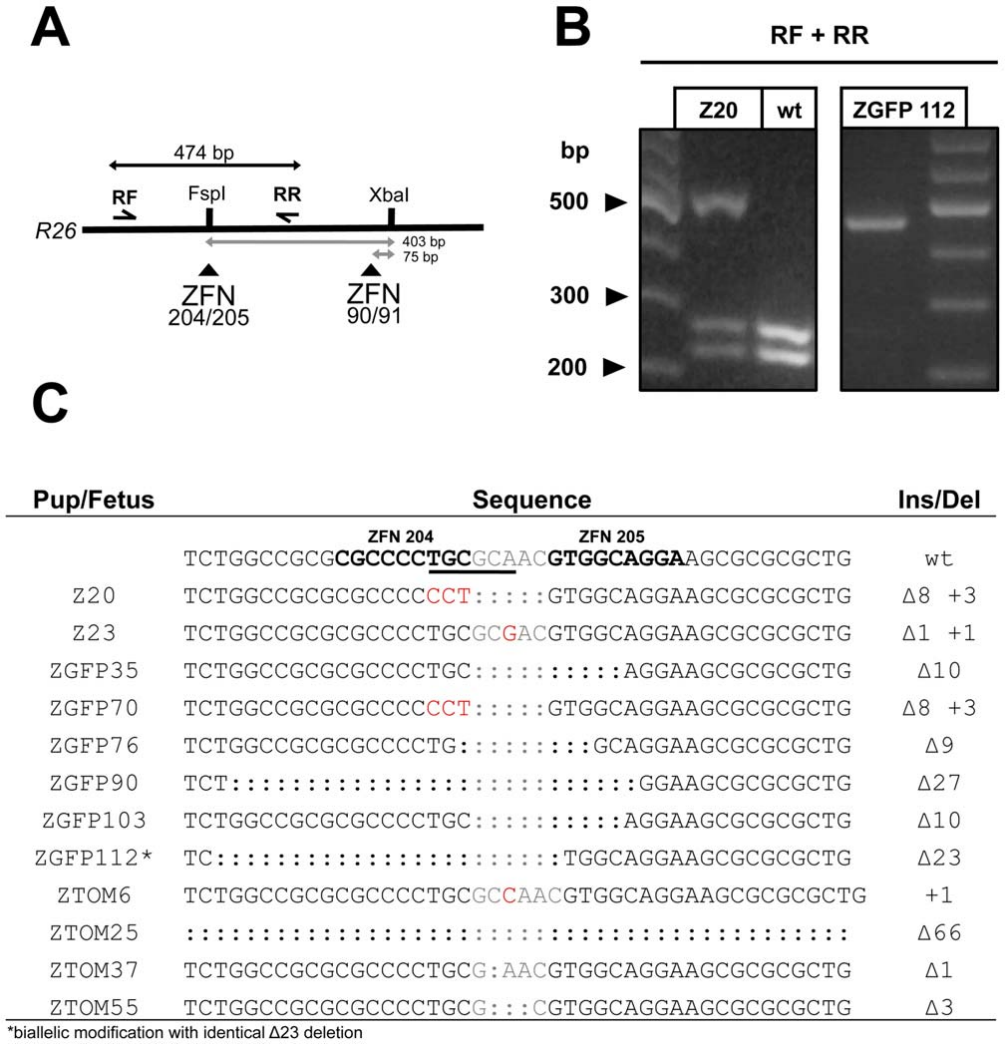
Non-homologous end joining repair of ZFN-generated double-strand breaks within the *ROSA26* locus. (A) Schematic of ZFN 90/91 and 204/205 target sites within *ROSA26* intron 1. ZFN pairs 90/91 and 204/205 target sites 75 bp and 403 bp upstream of the *Xba*I site (white arrows), which is routinely used in *ROSA26* targeting, respectively. ZFNs 204/205 target a partial *Fsp*I recognition sequence. RF and RR, *ROSA26* forward and reverse primers used for NHEJ analysis generating a 474 bp fragment (black arrows). (B) Screening for NHEJ repair at the ZFN204/205 cleavage site. Genomic DNA extracted from fetuses or pups developing from ZFN-injected zygotes was amplified with primers RF and RR and subjected to *Fsp*I restriction digest. Most error-prone NHEJ repair events eliminate the *Fsp*I recognition sequence (underlined in C) resulting in an indigestible band at 474 bp. In the majority of founders such as Z20 both modified and wt alleles were detected, however only mutated alleles were present in founder ZGFP112. (C) Cloning and sequencing of undigested PCR products reveals mutations around the ZFN204/205 cleavage site. Founder ZGFP112 carried an identical Δ23 deletion in both *ROSA26* alleles. ZFN 204/205 recognition sites highlighted in bold and the spacer region in grey color.

The mouse *ROSA26* locus is a “safe harbour” frequently used for site-specific insertion of transgenes by HR. Previous studies have demonstrated the feasibility of gene targeting in the *ROSA26* locus by use of commercially available ZFNs [28]. In a complementary approach we wanted to investigate whether OPEN ZFNs also allow modification of the *ROSA26* locus. Due to constraints in the targeting range of the OPEN system it was, however, not possible to target a ZFN pair directly to the *Xba*I site in the *ROSA26* locus that is frequently used to insert transgenes. Instead, two ZFN pairs were used in our experiments that could mediate DNA cuts in proximity of this *Xba*I site. These ZFN pairs, 90/91 and 204/205, target the *ROSA26* sequence 75 and 403 bp upstream of the *Xba*I site, respectively (Figure 1A, Table S1).

Initially, we injected ZFN pairs as *in vitro* synthesised mRNA into the cytoplasm of zygotes. Specific ZFN activity was estimated by the number of imprecise NHEJ events in the genome of the resulting offspring. Cytoplasmic microinjection of mRNAs encoding the 90/91 heterodimeric pair did not result in any discernable ZFN activity either in the form of mutagenic NHEJ or through HR upon co-injecting the pRosa26.8 donor construct [28] (Table 1) that induces alterations at the *Xba*I site 75 bp downstream of the 90/91 cleavage site. Also cytoplasmic microinjection of mRNAs encoding the 204/205 homodimeric ZFN pair at a concentration of 10 ng/µl appeared to be toxic and did not result in genome modification through NHEJ. In addition, we observed high toxicity but no NHEJ or HR after co-injections of the same mRNAs with targeting vector gtR26_EGFP containing an EGFP expression cassette sized 3.5 kb and equipped with 1.4 and 1.8 kb long homology arms flanking the ZFN recognition site (data not shown). This toxicity also persisted upon co-injecting reduced concentrations (2 ng/µl) of 204/205 homodimeric mRNAs. Thus, none of the experiments performed with the heterodimeric pair 90/91 and homodimeric pair 204/205 led to any discernable activity in mouse zygotes.

**Table 1:** Compilation of zygote microinjection experiments.

ZFN pair	Vectors	Cargo Type	Cargo Conc. ng/µl	Integration substrate	Substrate Conc. ng/µl	zygotes injected/transferred	born (% of transferred)	Phenotype	Mutated (% of F0)
90/91	MLM290/292 heterodimers	mRNA	10+10	pRosa26.8* linear **TV**	5	594/345	30 (8.7)	-	-
204/205	pST1374 homodimers	mRNA	10+10	-	-	123/36	8 (2.2)	-	-
204/205	pST1374 homodimers	mRNA	10+10	gtR26_EGFP linear **TV**	4	705/382	4 (0.1)	-	-
204/205	pST1374 homodimers	mRNA	2+2	gtR26_EGFP linear **TV**	1	1243/711	51 (7.2)	-	-
204/205	MLM290/292 heterodimers	mRNA	10+10	-	-	358/192	27 (14.1)	NA	2 NHEJ (7.4)
204/205	MLM290/292 heterodimers	mRNA	10+10	gtR26_EGFP linear **TV**	5	585/256	51 (20.0)	16 EGFP+	1 HR
2 NHEJ (3.9)									
204/205	MLM290/292 heterodimers	mRNA	10+10	gtR26_EGFP sc **TV**	10	275/195	46 (23.6; E)	2 EGFP+	4 NHEJ (8.7)
204/205	MLM290/292 heterodimers	mRNA	10+10	gtR26_tdT linear SA **TV**	5	640/287	87 (30.3; E)	12 tdT+	4 NHEJ (4.6)
						**4523/2404**	**304**		

EGFP: enhanced green fluorescent protein, NHEJ: non-homologous end-joining, TV: targeting vector, HR: homologous recombination, sc: supercoiled, tdT: tdTomato, SA: splice-acceptor, conc: concentration; E: 15dpc embryos,

* pRosa26.8 described in [28].

Failing to observe any activity with ZFN configurations described above we proceeded by using heterodimeric versions of the 204/205 ZFN pair. Cytoplasmic injection of mRNAs encoding heterodimeric 204/205 ZFNs was well tolerated by the embryos and led to efficient disruption of the ZFN 204/205 target sequence in a total of 12 founder animals as detected by *Fsp*I digestion and confirmed through sequence analysis (Figure 1B,C). Founders carrying NHEJ-mediated disruption were consistently obtained across several injection sessions (Table 1). One of the injection series yielded a founder in which both alleles of the *ROSA26* locus had been mutated. These alleles, which could be discriminated by a C/T SNP 33 bp upstream of the ZFN cleavage site, contained identical 23 bp deletions (Figure 1A,B, Figure S1B).

**Figure 2:**
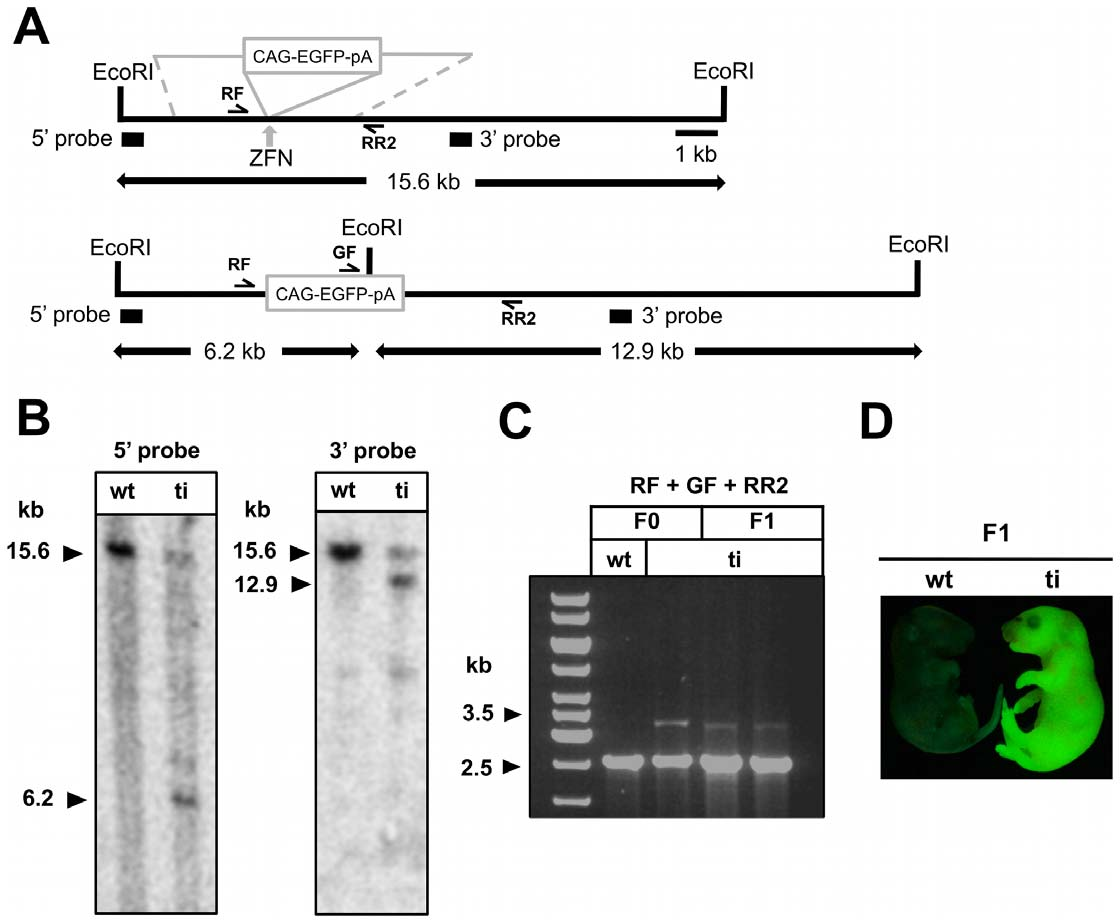
ZFN 204/205 promote *ROSA26* targeting by homologous recombination in mouse zygotes. (A) HR targeting strategy for the insertion of the targeting vector gtR26_EGFP carrying EGFP driven by a CAG promoter into the *ROSA26* locus. (B) Southern blot analyses of *Eco*RI digested genomic DNA from a GFP-fluorescent animal showing site-specific integration into the *ROSA26* locus. Both 5′ and 3′ probes detect only one expected fragment in the DNA of wild-type (wt) animal. Additional fragments detected in the DNA of targeted animal (ti) are consistent with the integration of the CAG-EGFP cassette into one of the *ROSA26* alleles. (C) Germline transmission of the *ROSA26-CAG-EGFP* allele was confirmed by junction PCR in two F1 mice, one of which is depicted in (D). Primers RF, GF, and RR2 generate a 2.5 kb fragment from *ROSA26* wt alleles, while an additional 3.2 kb fragment is amplified from a gtR26_EGFP targeted allele.

To investigate the possibility of using the 204/205 ZFN pair to induce HR at the ZFN cleavage site, we co-injected the ZFN mRNAs with a linear DNA fragment of the targeting vector gtR26_EGFP. We identified 16 fluorescent founder animals and could confirm that one of them carried the gtR26_EGFP cassette correctly integrated into the ZFN target site as confirmed by Southern blot analysis (Figure 2B) as well as by junction PCR and sequencing (Figure 2B, S2). The integrated EGFP transgene could be passed to the next generation and remained active in F1 offspring (Figure 2C,D). In further experiments we co-injected the full-length super-coiled gtR26_EGFP targeting vector, since vectors with super-coiled topology served as efficient donors in previous studies [28], [29]. We failed, however, to observe HR in this particular experiment (Table 1).

To test whether integration at the ZFN 204/205 cleavage site would allow transgene expression under the same transcriptional control as has been reported before for *ROSA26* insertion transgenes, we co-injected the linear targeting construct gtR26_tdT carrying a 2.5 kb splice-acceptor tdTomato cassette. Despite identifying 12 pups that expressed tdTomato and several others carrying independent NHEJ events, we could not identify any animals with homologous integration of the gtR26_tdT vector as determined by Southern blot and junction PCR even though NHEJ events were present (table 1, data not shown). This result shows that despite ZFN activity, the gtR26_tdT expression cassette did not integrate into the *ROSA26* locus. This observation was surprising because the gtR26_tdT vector included identical homology arms and a smaller insert compared to gtR26_EGFP. Therefore, we conclude that the 12 tdTomato expressing mice are most likely the result of random transgene integration and a partially active *ROSA26* promoter that was included in the left homology arm of the gtR26_tdT construct.

## Discussion

Nuclease-assisted gene targeting in zygotes offers a more expeditious alternative when compared to standard gene targeting in mouse ES cells. This becomes particularly important for frequently targeted loci such as *ROSA26*. An additional advantage comes with the species and strain-independent cleavage mechanism of ZFNs, which allows generation of germline-competent founders in all organisms accessible to embryo manipulation [32]. We explored the potential of OPEN ZFNs as an alternative for targeted transgenesis in mouse embryos and conclude that OPEN ZFN mRNAs can be used to engineer the mouse genome by direct zygote injection.

Injection statistics compiled in Table 1 clearly show that in the case of zygotes expressing the *ROSA26* ZFN 204/205 heterodimer pair NHEJ repair occurs more frequently than HR as described in earlier studies [28], [29]. NHEJ events observed in 4 to 9% of offspring with this single pair are well within the range of NHEJ frequencies observed in other studies using ZFNs from OPEN selection or the CoDA pool in human cells, zebrafish embryos or plants [12], [15], [19]. However, in rodent zygotes NHEJ modification rates above 20% were reported after microinjection of ZFNs obtained through the Sigma-Aldrich CompoZr service [27]–[29]. Mosaicism and the presence of two or more modified alleles in a single animal were frequently observed in these studies. We identified one founder, ZGFP112, carrying an identical deletion in both *ROSA26* alleles. This genotype could be the result of a primary NHEJ deletion in one allele which served as a homologous donor in the subsequent repair of the second DSB by HR. Alternatively, microhomology domains (Figure S1B) in proximity to a DSB could have triggered a preferential mode of end-joining leading to identical outcomes of individual repair events [27]. Whether the lower ZFN activity rates in our study as compared to previous studies are the result of locus-dependent effects, differences in ZFN binding activity, variations in injection procedures, or lack of codon optimization in our ZFN expression vectors for expression in mammalian cells will only be revealed by further comparative studies. Also, a recent study showed that, at least in cultured cells, the cleavage activity of *ROSA26-*specific ZFNs generated by modular assembly [33] increased significantly upon incorporation of additional ZF modules. However, we note that this strategy does not always increase activity and care must be taken with the choice of linkers used to add more fingers because these more extended ZFNs can potentially bind to a greater range of off-target sites using subsets of fingers [10].

Meyer and colleagues reported successful vector integration into *ROSA26* in 1.7% to 4.5% of pups born. In our study, HR-mediated modification of the *ROSA26* was observed in a single germline-competent founder out of 51 mice born after co-injecting 585 zygotes with ZFN 204/205 mRNA and the linear gtR26_EGFP donor construct. Surprisingly, no targeted integration was detected when supercoiled gtR26_EGFP, the preferred donor topology used in earlier studies [28], [29], or linear gtR26_tdT, a targeting vector with identical homology arms, were co-injected with ZFNs 204/205. Thus, a total of 1500 zygotes were injected with ZFNs 204/205 together with donor constructs to yield a single targeted founder. While we cannot formally exclude that the observed targeted integration event results from the resolution of a spontaneously occurring DSB and is unrelated to ZFN activity, this scenario seems rather unlikely in light of previously published data. To date, only one study has ever reported spontaneous homologous integration of a targeting construct into the genome of microinjected zygotes and more than 10,000 zygotes were injected to obtain a single targeting event in that report [34].

The issue of ZFN toxicity has often been raised as an indicator for off-target ZFN activity and thus a potential limitation of the technology. Although the mice generated in this study were not tested for off-target cleavage events, we have not observed a marked increase in embryo lethality upon injection of any heterodimeric ZFNs as compared to conventional pronuclear injections routinely performed in our laboratory. This is in stark contrast to microinjections of homodimeric ZFNs, which caused significant embryo lethality most likely due to more frequent off-target cleavage events [35].

Thus far, the influence of ZFN activity on gene targeting efficiency has not been studied comprehensively in microinjected embryos. However, earlier studies [28], [29] suggest a positive correlation between the number of NHEJ repair events and HR-mediated targeting events, which both depend on the frequency of DSBs and ultimately on ZFN activity. The size of the desired modification is clearly another factor influencing targeting efficiency with small modifications such as adding or replacing a small number of nucleotides clearly faring better than experiments requiring integration of large inserts at the same locus [29].

In our hands only one out of the two *ROSA26* OPEN ZFN pairs showed both NHEJ and HR activities in mouse zygotes. Recently described novel reporter systems translating nuclease-induced frameshift events into a switch between two discrete fluorescent signals [36], [37] may be useful to quickly identify active ZFNs in cultured cells prior to commencing the actual gene targeting experiments in zygotes. In addition to ZFNs, the recently described TAL Effector Nucleases (TALENs) show promise as an alternative method for rapid assembly of site-specific nucleases [38]–[42], but have to still prove their potential for use in mouse oocytes.

Based on the experiments presented here, OPEN ZFNs represent viable tools for achieving NHEJ-mediated gene knockout in mouse zygotes. Since we observed only one event, we cannot estimate the efficiency of OPEN 204/205 ZFN heterodimers for achieving integration of a targeting vector into the *ROSA26* locus. Only further experiments will reveal how OPEN ZFNs compare in general with other systems in supporting HR in mouse zygotes.

## Materials and Methods

### Animals

Females and males of BDF1 (B57BL/6×DBA/2), C57BL/6, and CD1 mice were purchased from a commercial breeder (Charles River, Germany). All animals were maintained in temperature- and light-controlled rooms (12 light/12 dark, light on from 6:00 a.m.) with food and water *ad libidum*. All experiments including laboratory animals were approved by the Cantonal Veterinary Office of Zurich. The protocol of animal handling and treatment was in accordance with Swiss Federal and Cantonal regulations as well as the internal guidelines of the University of Zurich.

### Embryo Collection, Culture and Manipulation

B6D2F1 or C57BL/6 female mice underwent ovulation induction by intra peritoneal (i.p.) injection of 5 IU pregnant mare's serum gonadotrophin (PMSG; Folligon – InterVet, Switzerland), followed by i.p. injection of 5 IU human chorionic gonadotropin (hCG; Pregnyl – Essex Chemie, Switzerland) 48 h later. For the recovery of zygotes, the B6D2F1 and C57BL/6 females were mated with the males of the same strain immediately after the administration of hCG. All zygotes were collected from oviducts 24 h after the hCG injection and were then freed from any remaining cumulus cells by a 1–2 min treatment of 0.1% hyaluronidase (Sigma) dissolved in M2 medium.

Mouse embryos were cultured in M16 (Sigma) medium at 37°C and 5% CO_2_. For micromanipulation the embryos were transferred into M2 medium (Sigma).

### Cytoplasmic and pronuclear microinjections

All microinjections were performed using a microinjection system comprised of an inverted microscope equipped with Nomarski optics (Nikon, Japan), set of micromanipulators (Narashige, Japan) and a FemtoJet microinjection unit (Eppendorf, Germany). ZFN mRNAs were injected into the cytoplasm whereas the DNA expression constructs and DNA targeting fragments were injected into the male pronuclei; in experiments where mRNA and DNA were co-injected the RNA DNA mixture was first injected into the male pronucleus and subsequently into the cytoplasm upon the withdrawal of the microinjection capillary. Specific concentrations of injected mRNAs and DNA constructs are compiled in Table 1.

### Embryo Transfer

Embryos that survived the microinjection were transferred on the same day into the oviducts of 8–16 weeks old pseudopregnant CD-1 females (0.5 days post coitus) that have been mated with sterile TgV males [43] on the day before embryo transfer. Pregnant females were allowed to deliver and raise their pups or were sacrificed at 14–16 days post embryo transfer so that the developing foetuses could be removed for analysis.

### Construction of ZFN expression vectors and mRNA preparation

Zinc finger proteins binding target sites 75 and 403 bp upstream of the *Xba*I site within the *ROSA26* intron 1 were selected using the previously described OPEN method [12]. Selected zinc fingers (Text S1) were cloned as *Xba*I-*BamH*I fragment into either the expression vectors pST1374 or pMLM290/pMLM292 that express homo- or heterodimeric ZFNs, respectively [35]. In both ZFN expression vectors, the CMV promoter was replaced by a CMV early enhancer element/chicken beta-actin promoter (CAG) promoter [44].

*In vitro* mRNA transcription, capping and polyadenylation, was performed using the mMESSAGE mMACHINE T7 Ultra Kit. Prior to injection the mRNAs were purified using the NucAway Spin Columns (Ambion). mRNA quality was verified by denaturing gel electrophoresis and concentration was quantified using spectrophotometry.

### Construction of targeting vectors

Targeting vector GTR26 includes a 1.4 kb 5′ *ROSA26* homology arm and a 1.8 kb 3′ *ROSA26* homology arm flanking a central *Swa*I restriction site. An expression cassette consisting of a 1.6 kb CAG promoter/enhancer followed by the 720 bp EGFP coding region and the 531 bp rabbit beta-globin polyadenylation site (3.5 kb in total, including 5′ and 3′ flanking sequences) was inserted by blunt cloning into the *Swa*I site to generate targeting vector GTR26-EGFP. To generate targeting vector GTR26-tdT a cassette including the 104 bp Ad2 splice-acceptor followed by a 590 bp triple-STOP-pCMV-IRES fragment, the 1.4 kb tdTomato coding region and the 256 bp TK polyadenylation signal (2.5 kb in total, including 5′ and 3′ flanking sequences) was PCR-amplified from pXLBluescriptII PTS tdTomato (gift of J. Ruiz and K. Rector) using primers AGG GCG CAG TAG TCC AGG GTT TCC and GGC TAT GGC AGG GCT TGC CGC C with *Pfu* polymerase and cloned into the SwaI site of the GTR26 targeting vector. To generate a linear fragment all GTR26^−^based targeting vectors were *Pac*I digestion prior to microinjection.

### NHEJ and Targeted integration detection assays

Genomic DNA was extracted from mouse biopsies or fetal tissue using a buffer containing 10 mM Tris-HCl pH 9, 50 mM KCl, 0.45% Nonident p40, 0.45% Tween 20 and Proteinase K. Extracts were subjected to Phenol/Chloroform/Isoamyl alcohol purification, precipitated with Isopropanol, and dissolved in EB buffer (Qiagen).

For detecting NHEJ repair at the *ROSA26* locus, primers RF (GCC GCC CAC CCT CCC CTT CCT C) and RR (CGC CTA CT CCA CTG CAG CTC CC) were used to amplify a 474 bp fragment surrounding the ZFN204/205 target site. 25 µl of each PCR product were digested with *Fsp*I and subsequently resolved on a 2% agarose gel. Samples including undigested PCR fragments were cloned into pGEM-T easy (Promega) for Sanger sequencing.

Targeted integration of donor vectors was assessed by junction PCR and Southern blotting. In case of ZFN204/205 –mediated *ROSA26* targeting primers GF (GCC GGG ATC ACT CTC GGC ATG) and RR2 (CAC CAC TGG CTG GCT AAA CTC TGG) amplified the 3′ junction that is specific for the integration of GTR26-204/205-CAG-EGFP into the mouse *ROSA26* locus. For Southern Blot analysis 10 µg of genomic DNA were digested overnight at 37°C with *Eco*RI, resolved on a 0.7% agarose gel, and transferred to nylon membranes. Membranes were heat-fixated at 65°C for 1 h and incubated with prehybdrization solution as described [45] over night at 65°C. The Rosa 26 5′ probe, a 695 bp *Eco*RI/*Pac*I fragment, was generated from the “Orkin” plasmid. The *ROSA26* 3′ probe, a 615 bp, *Eco*RI fragment, was generated from plasmid pCRII-Rosa 3′. Hybridization probes were heat denatured, labeled with P^32^ marked dCTP (Perkin Elmer) using the Ladderman Labelling Kit (Takara). The labeled probe was purified with illustra MicroSpin S-200 HRcolumns (GE Healthcare) and heat-denatured probe in hybridization buffer was added to the membranes for overnight rotation at 65°C. Membranes were washed three times (5 min) using 2× SSC. The membranes were exposed at room temperature for 1–3 days and imaged using a Storm 840 phospho-imager (Molecular Dynamics). Digital images of Southern Blots were processed with ImageJ.

## Supporting Information

Figure S1 **Sequencing of ZFN204/205 cleavage site within** ***ROSA26*** **locus.** (A) Sequencing traces for NHEJ-modified *ROSA26* alleles. One representative trace per founder is shown. (B) NHEJ-modified alleles in founder ZGFP112. A deletion of 23 bp around the ZFN204/205 cleavage site could be identified in both *ROSA26* alleles in founder ZGFP112. The presence of a C/T SNP (red arrow) 33 bp upstream of the ZFN cleavage site (underlined in red) in this founder enabled the identification of individual *ROSA26* alleles. Possible regions of microhomology, which can attract NHEJ repair and increase the likelihood of certain NHEJ repair outcomes are underlined in black.
(TIF)

Figure S2 **Sequencing of junction PCR product amplified from a founder carrying a targeted** ***ROSA26*** **allele.** The upper panel shows the parts being sequenced with (1) covering parts of the EGFP open reading frame and the polyadenylation signal and (2) covering the junction of *ROSA26* genomic DNA and the 3′ homology arm of integrated targeting construct GTR26_EGFP.
(TIF)

Table S1 **Sequences of ZFN target sites.** Capital letters denote Zinc finger module binding sequences, bold letters highlight binding to the parallel or antiparallel strand, respectively.
(PDF)

Text S1 **Sequences of OPEN Zinc Finger modules used in this study.**
(PDF)
