# Supplementary material for: Evaluation of OPEN Zinc Finger Nucleases for Direct Gene Targeting of the ROSA26 Locus in Mouse Embryos
Source: PLoS One. 2012 Sep 6;7(9):e41796. doi: 10.1371/journal.pone.0041796 (PMC3435328; doi:10.1371/journal.pone.0041796)
Supplement: Table S1 — Sequences of ZFN target sites. Capital letters denote Zinc finger module binding sequences, bold letters highlight binding to the parallel or antiparallel strand, respectively. (PDF) [file pone.0041796.s003.pdf]

Gene	ZFN Name	Site Sequence
R26	91	cTGCTGCCTCctggct**TCTGAGGAC**c
	90	g**ACGACGGAG**gaccgaAGACTCCTGg
R26	204	gCGCCCCTGCgcaac**GTGGCAGGA**a
	205	c**GCGGGGACG**cgttgCACCGTCCTt
