# Supplementary material for: Evaluation of OPEN Zinc Finger Nucleases for Direct Gene Targeting of the ROSA26 Locus in Mouse Embryos
Source: PLoS One. 2012 Sep 6;7(9):e41796. doi: 10.1371/journal.pone.0041796 (PMC3435328; doi:10.1371/journal.pone.0041796)
Supplement: Text S1 — Sequences of OPEN Zinc Finger modules used in this study. (PDF) [file pone.0041796.s004.pdf]

>ZFN90
TCTAGACCCGGGGAGCGCCCCTTCCAGTGTCGCATTTGCATGCGGAACTTTTCGAGCATGGAGCAGCTTGCCAGGCATACCCGTACTCATAC
CGGTGAAAAACCGTTTCAGTGTCGGATCTGTATGCGAAATTTCTCCCAGACGGAGACGTTGAAGAGGCATCTACGTACGCACACCGGCGAG
AAGCCATTCCAATGCCGAATATGCATGCGCAACTTCAGTCGCGCCGACAACCTGCGCAGGCACCTAAAAACCCACCTGAGGGGATCC

>ZFN91
TCTAGACCCGGGGAGCGCCCCTTCCAGTGTCCCATTTGCATGCGGAACTTTTCGGACGAGGCGAACCTTAGGCGCCATACCCGTACTCATAC
CGGTGAAAAACCGTTTCAGTGTCGGATCTGTATGCGAAATTTCTCCCGCGCGGACAACTTGGGCAGGCATCTACGTACGCACACCGGCGAG
AAGCCATTCCAATGCCGAATATGCATGCGCAACTTCAGTGCGCGGAACACGCTGAAGGGCCACCTAAAAACCCACCTGAGGGGATCC

>ZFN204
TCTAGACCCGGGGAGCGCCCCTTCCAGTGTCGCATTTGCATGCGGAACTTTTCGGACAACGCCCACCTTGCGCGCCATACCCGTACTCATAC
CGGTGAAAAACCGTTTCAGTGTCGGATCTGTATGCGAAATTTCTCCCAGACCGAGACCTTGAAGAGGCATCTACGTACGCACACCGGCGAGA
AGCCATTCCAATGCCGAATATGCATGCGCAACTTCAGTAGGCCCGACGCCCTGACCCGCCACCTAAAAACCCACCTGAGGGGATCC

>ZFN205
TCTAGACCCGGGGAGCGCCCCTTCCAGTGTCGCATTTGCATGCGGAACTTTTCGAAGCGCCACACGCTTACCCGCCATACCCGTACTCATAC
CGGTGAAAAACCGTTTCAGTGTCGGATCTGTATGCGAAATTTCTCCCGGCGCGAGCACTTGGTGCGCCATCTACGTACGCACACCGGCGAGA
AGCCATTCCAATGCCGAATATGCATGCGCAACTTCAGTCAGACGGCCACCCTGAAGCGGCACCTAAAAACCCACCTGAGGGGATCC
